# Epicardial Adipose Tissue Thickness in Patients With Subclinical Hypothyroidism and the Relationship Thereof With Visceral Adipose Tissue Thickness

**DOI:** 10.14740/jocmr2460w

**Published:** 2016-01-26

**Authors:** Dilek Arpaci, Aysel Gurkan Tocoglu, Sabiye Yilmaz, Sumeyye Korkmaz, Hasan Ergenc, Huseyin Gunduz, Nurgul Keser, Ali Tamer

**Affiliations:** a Department of Endocrinology and Metabolism, Faculty of Medicine, Bulent Ecevit University, Zonguldak, Turkey; b Department of Internal Medicine, Faculty of Medicine, Sakarya University, Sakarya, Turkey; c Department of Cardiology, Faculty of Medicine, Sakarya University, Sakarya, Turkey

**Keywords:** Epicardial adipose tissue, Adipose tissue thickness, Subclinical hypothyroidism

## Abstract

**Background:**

Subclinical hypothyroidism (SH) is associated with cardiovascular metabolic syndromes, especially dislipidemia and abdominal obesity. Visceral abdominal adipose tissue (VAAT) and epicardial adipose tissue (EAT) have the same ontogenic origin and produce many proinflammatory and proatherogenic cytokines. We evaluated EAT and VAAT thickness in patients with SH.

**Methods:**

Forty-one patients with SH and 35 controls were included in the study. Demographical and anthropometric features of both patients and controls were recorded. Thyroid and metabolic parameters were measured. EAT was measured using 2D-transthoracic echocardiography.

**Results:**

The age and gender distributions were similar in the two groups (P = 0.998 and P = 0.121, respectively). Body mass index (BMI), fat mass, waist circumference (WC), hip circumference (HC), the WC/HC ratio, and the thicknesses of VAAT and abdominal subcutaneous adipose tissue were higher in the case group than the control group (all P values < 0.01). However, both groups had similar EAT thickness (P = 0.532), which was positively correlated with BMI, fat mass, WC, HC, VAAT thickness, abdominal subcutaneous adipose tissue thickness, and serum triglyceride (TG) level (all P values < 0.01). We found no correlation between EAT thickness and thyroid-stimulating hormone (TSH) level, free thyroxine (FT4) level, or low-density lipoprotein-cholesterol (LDL-C) level, and anti-TPO level (all P values > 0.05). We found no difference between the two groups in fasting plasma glucose (FPG) level (P = 0.780), but the levels of LDL-C and TG differed significantly (P = 0.002 and P = 0.026, respectively). The serum TSH level was higher and the FT4 level was lower in the case than the control group (both P values <0.01).

**Conclusion:**

Increased abdominal adipose tissue thickness in patients with SH is associated with atherosclerosis. To detemine the risk of atherosclerosis in such patients, EAT measurements are valuable; such assessment is simple to perform.

## Introduction

Subclinical hypothyroidism (SH) is considered present when the thyroid-stimulating hormone (TSH) level is high but the levels of free thyroxine (FT4) and free tri-iodothyronine are normal. The most common cause of overt hypothyroidism and SH is Hashimoto’s thyroiditis in iodine sufficient countries. The prevalence of SH in general populations is 4-20% and in women older than 60 years is up to 20% [1, 2]. Various reports have suggested that patients with overt hypothyroidism may exhibit risk factors for cardiovascular disease, including abdominal obesity, metabolic syndrome, dyslipidemia, diastolic hypertension, hypercoagulability, and endothelial dysfunction. However, any relationship between SH and atherosclerosis remains controversial [3, 4]. A relationship between SH and the presence of cardiovascular disease (CVD) has been reported in large population-based studies [5-7]. Even in SH patients with high-level TSH, an increased visceral abdominal adipose tissue (VAAT) thickness has been shown to be an independent risk factor for CVD [5, 8].

VAAT surrounds the internal organs, and increased levels of VAAT are more significant than increased levels of subcutaneous fat in terms of the risks of metabolic syndrome, insulin resistance, and cardiovascular mortality [9]. Epicardial adipose tissue (EAT) is located between the myocardium and visceral epicardium. EAT and VAAT have the same ontogenic origin [10]. This is important, because both of these fat tissues produce many proinflammatory and proatherogenic cytokines [11, 12].

EAT thickness was determined as the accumulation of visceral adipose around the heart [11]. Increased evidence showed that EAT can directly regulate vasomotor function by diffusion of its bioactive molecules into the nearby intima-media layer of the vessel wall [12-14]. EAT is a new, non-invasive method of the measurement for early detection of predisposition to atherosclerosis [4]. In most diseases (e.g. hypertension, CVD, and metabolic syndrome), a relationship between EAT and atherosclerosis was determined [15-20].

A few studies found that EAT thickness was elevated in patients with SH [13-15]. However, only one report to date has explored the relationship between EAT and VAAT [16]. The present study sought to determine whether the levels of VAAT and EAT in patients with SH differed from those of healthy control subjects.

## Materials and Methods

### Selection of subjects

The present study included 41 patients with SH and 35 healthy controls (matched in terms of age and gender). All participants signed informed consent forms. The study was approved by the Ethics Committee of Sakarya University Faculty of Medicine (24.02.2014, No. 28). Patients with positive anti-TPO and with chronic thyroiditis findings on ultrasonography were eligible for the study. Patients group was selected by following criteria: who had serum TSH level > 5.4 IU/mL with normal FT4, not any medication effect thyroid status and not any disorder without hypothyroidism. Control subjects were selected from the normal population in staff of our hospital such as nurse, secretary or doctors who had no history or laboratory abnormalities. Demographic data on patients and controls were recorded.

### Exclusion criteria

The exclusion criteria were a history of smoking, diabetes, or hypertension, non-Hashimoto thyroiditis (a systemic disease), CVD, secondary or post-surgical hypothyroidism, pregnancy, age < 18 or > 60 years, and/or any active infection.

### Measurements

Body weight and height were measured without shoes using a wall-mounted stadiometer and the body mass index (BMI) calculated (in kg/m^2^). Waist circumference (WC) was measured at the mid-point between the iliac crest and the lower rib. Hip circumference (HC) was measured at the level of maximal gluteal protrusion.

### Bodily parameters

The basal metabolic rate and body fat percentage of each subject were evaluated using a Tanita Body Composition Analyzer (Model TBF-300; Tanita Corporation, Tokyo, Japan) with each subject in a standing position, without shoes, wearing light clothing, after a ≥ 8-h fast, under conditions of adequate hydration. Abdominal subcutaneous adipose tissue thickness and VAAT thickness were recorded using a Tanita Abdominal Fat Analyzer (AB-140 Viscan; Tanita Corporation) (which measures bioelectrical impedance). Blood pressure was measured after at least 10 min of rest using a sphygmomanometer (ERKA; Bad Tolz, Germany). Two measurements were taken and the average was calculated.

### Biochemical analysis

Venous blood samples were collected in the morning after a 12 h fasting. We measured the levels of fasting plasma glucose (FPG), low-density lipoprotein-cholesterol (LDL-C), high-density lipoprotein-cholesterol (HDL-C), triglyceride (TG), FT4, and TSH. Serum TSH and FT4 levels were measured using chemiluminescence-based methods. The normal level of FT4 was taken to be 9 - 19 pmol/L and that of TSH 0.35 - 5.4 µU/mL. Serum glucose levels were measured using an enzymatic (hexokinase) method (Roche Diagnostics GmbH). HDL-C levels were measured using a selective detergent and TG levels employing a colorimetric assay featuring xylidyl blue (Roche Diagnostics GmbH; Mannheim, Germany). LDL-C levels were determined using the Friedewald formula.

### Echocardiography

All patients visited the Department of Cardiology at Sakarya Training and Research Hospital, and EAT thickness was measured (using 2D-transthoracic echocardiography) by the same cardiologist. Parasternal long- and short-axis EAT images, which permit the most accurate measurements on the right ventricle, were obtained using a standard technique with each subject in the left lateral decubitus position. The EAT thickness was defined as the depth of the echo-free space between the outer wall of the myocardium and the visceral layer of the pericardium, at end-systole of the right ventricle [17].

### Statistical methods

Statistical analyses were performed with the aid of SPSS version 18.0 (SPSS Inc., Chicago, IL). Numerical variables are shown as mean ± standard deviation (SD). Categorical data are expressed as numbers with percentages. Categorical between-group differences were compared using the Chi-squared test. One-way ANOVA was used to compare data from the case and control groups. Continuous variables are expressed as either mean ± SD or as medians (maxima minus minima), and categorical variables are expressed as either frequencies or percentages. Continuous variables were compared using the independent-samples *t*-test or the Mann-Whitney U-test, and categorical variables were compared using Pearson’s Chi-squared test. A P value < 0.05 was considered to indicate statistical significance.

## Results

We studied 41 patients with SH (cases) and 35 healthy subjects (controls). The two groups did not differ in terms of gender or age. Of all subjects, 95% (n = 39) were female and 5% (n = 2) were male in the case group; all controls were female (P = 0.998). Mean age was 34.07 ± 6.70 years in case group and 31.82 ± 5.57 years in control group (P = 0.121). BMI, fat mass, WC, WC/HC ratio, and thicknesses of VAAT and abdominal subcutaneous adipose tissue were higher in the case group than in the control group. Although FPG levels did not differ between the two groups (P = 0.780), the LDL-C and TG levels did differ significantly (P < 0.01 and P = 0.026, respectively). The serum TSH and anti-TPO levels were higher in the case group than in the control group (P < 0.01). The FT4 level was lower in the case group than in the control group (P < 0.01). While there were significant differences between BMI, fat mass, WC, WC/HC ratio, LDL-C, and TGs, there were no differences in EAT between two groups. Mean EAT was 4.61 ± 0.06 cm in case group and 4.51 ± 0.07 cm in control group (P = 0.532) (Table 1).

**Table 1:** A Comparison of Demographic and Laboratory Data Between the Two Groups

Feature	Case (n = 41)	Control (n = 35)	P value
Age (years)	34.07 ± 6.70	31.82 ± 5.57	0.121
Gender (F/M)	39/2	35/0	0.998
BMI (kg/m^2^)	27.84 ± 5.26	23.72 ± 3.01	< 0.01
Fat mass (%)	31.74 ± 7.58	26.80 ± 6.42	< 0.01
Fat mass (kg)	23.09 ± 9.89	16.85 ± 5.99	< 0.01
Waist circumstance (WC, cm)	93.68 ± 12.09	87.94 ± 8.38	0.038
Hip circumstance (HC, cm)	103.86 ± 10.37	100.71 ± 6.73	0.152
WC/HC	0.95 ± 0.07	0.90 ± 0.005	< 0.01
Visceral adipose tissue	9.53 ± 4.03	7.10 ± 2.40	< 0.01
Abdominal subcutaneous adipose tissue	40.24 ± 6.57	35.33 ± 6.32	< 0.01
Systolic BP (mm Hg)	120.10 ± 7.24	118.97 ± 7.36	0.471
Diastolic BP (mm Hg)	62.23 ± 5.80	60.70 ± 3.89	0.065
FPG (mg/dL)	90.68 ± 6.30	89.12 ± 7.68	0.780
TSH (µIU/mL)	14.55 ± 6.50 (median 7.64 ± 1.55)	1.54 ± 0.82 (median 1.52 ± 0.78)	< 0.01
FT4 (pmol/L)	11.60 ± 2.68	14.15 ± 1.70	< 0.01
Anti-TPO (IU/mL)	313.65 ± 358.69	2.42 ± 1.16	< 0.01
LDL (mg/dL)	125.85 ± 31.73	104.39 ± 25.50	< 0.01
TG (mg/dL)	105.90 ± 50.08 (median 93.50 ± 4.96)	83.75 ± 27.17 (median 79.50 ± 2.50)	0.026
HDL (mg/dL)	55.21 ± 15.40	57.32 ± 11.79	0.495
EAT (mm)	4.61 ± 0.06	4.51 ± 0.07	0.532

EAT thickness was positively correlated with BMI (r = 0.402, P < 0.01), fat mass (r = 0.408, P < 0.01), WC (r = 0.400, P < 0.01), HC (r = 0.430, P < 0.01), VAAT thickness (r = 0.321, P < 0.01), abdominal subcutaneous adipose tissue thickness (r = 0.345, P < 0.01), and serum TG level (r = 0.270, P < 0.01) (Table 2). However, we found no correlation between EAT thickness and TSH (r = -0.094, P = 0.328), FT4 (r = 0.025, P = 0.794), or LDL-C (r = 0.028, P = 0.779) levels and anti-TPO level (Table 2).

**Table 2:** Pearson Correlation Analysis Between EAT and Other Parameters in the Whole Study Group

	Total (n = 76)	
	r value	P value
BMI (kg/m^2^)	0.402	0.000
Fat mass (kg)	0.408	0.000
WC (cm)	0.400	0.000
HC (cm)	0.340	0.001
Visceral fat mass (kg)	0.321	0.002
Abdominal fat mass (kg)	0.345	0.001
TSH (µIU/mL)	-0.094	0.328
FT4 (pmol/L)	0.025	0.794
Anti-TPO (IU/mL)	0.202	0.088
LDL-C (mg/dL)	0.028	0.779
TG (mg/dL)	0.270	0.006

Mean BMI was 27.84 ± 5.26 kg/m^2^ in case group and 23.72 ± 3.01 kg/m^2^ in control group (P = 0.000). Mean WC was 93.68 ± 12.09 cm in case group and 87.94 ± 8.38 cm in control group (P = 0.038). Mean HC was 103.86 ± 10.37 cm in case group and 100.71 ± 6.73 in control group (P = 0.152). WC/HC was 0.95 ± 0.07 in case group and 0.90 ± 0.05 in control group (P = 0.007).

Mean fat mass was 23.09 ± 9.89 kg in case group and 16.85 ± 5.99 kg in control group (P = 0.002). Visceral fat was 9.53 ± 4.03 kg in case group and 7.10 ± 2.40 in control group (P = 0.005). Abdominal fat was 40.24 ± 6.57 kg in case group and 35.33 ± 6.32 in control group (P = 0.004).

Mean FPG was 90.68 ± 6.30 mg/dL in case group and 89.12 ± 7.68 mg/dL in control group (P = 0.780). The mean LDL-C level was 125.87 ± 31.73 mg/dL in case group and 106.39 ± 25.50 mg/dL in control group (P = 0.002). The mean TGs level was 105.90 ± 50.08 mg/dLin case group and 83.75 ± 27.17 mg/dL in control group (P = 0.026).

TSH level was 14.55 ± 6.50 µIU/mL in case group and 1.54 ± 0.82 µIU/mL in control group (P = 0.000). FT4 was 11.60 ± 2.68 pmol/L in case group and 14.15 ± 1.70 pmol/L in control group (P = 0.000).

## Discussion

We found that SH patients had a higher BMI, a greater fat mass, and thicker abdominal fat and VAAT, as compared to controls matched in terms of age and gender. However, we failed to show differences in two groups for EAT. This study demostrated a correlation between EAT and BMI, fat mass, WC, HC and VAAT.

Hypothyroidism can cause weight gain. However, any association between TSH level and the amount of body fat remains controversial [8, 18, 19]. Abdominal obesity associated with increased VAAT thickness is an independent risk factor for CVD. An association between high-level TSH and increased VAAT thickness has been demonstrated [8]. WC is typically used as a measure of abdominal obesity. However, WC does not distinguish VAAT from abdominal subcutaneous adipose tissue. Magnetic resonance studies have shown that EAT thickness is a better indicator of VAAT thickness, independent of obesity status [14, 20]. A positive correlation between EAT thickness and VAAT thickness was evident; this correlation seemed to be more significant than the WC [20-22].

Akbaba et al [23] found that although VAAT thickness was higher in an SH group than controls, statistical significance was not attained. VAAT thickness decreased significantly after levothyroxine therapy. In contrast, we found that VAAT thickness was higher in SH patients than controls.

Although SH is usually incidentally diagnosed, an association between SH and atherosclerotic heart disease has been demonstrated. SH features some of the risk factors that can accelerate the development of atherosclerosis. Such risk factors include an increased BMI, greater VAAT thickness, insulin resistance, atherogenic dyslipidemia, hypercoagulability, and systolic and diastolic hypertension [24-26]. The Rotterdam study revealed a high frequency of cardiovascular events in elderly females with SH [5]. Also, a clear association was evident between both the presence and severity of CVD, and thyroid function [27].

Measurement of EAT thickness is a new, non-invasive technique. EAT is thought to cause atherogenesis, plaque instability, and neovascularization. This type of fat exerts paracrine/vasocrine effects and releases bioactive mediators [6, 28-30].

In previous studies, EAT thickness was greater in patients with SH than in healthy controls [13, 14, 16]. In contrast, although we found increased BMI, WC, HC, fat mass, abdominal subcutaneous adipose tissue, VAAT, LDL-C and TGs in SH patients than in the controls, we did not find any between-group difference.

Korkmaz et al [15] found that although EAT thickness was increased in patients with TSH levels > 10 IU/mL, no such association was evident in the general population. Our SH and control groups did not significantly differ in terms of EAT thickness. We found associations between EAT thickness and BMI, fat mass, and abdominal and visceral obesity, but no associations between EAT thickness and serum LDL-C, TSH, or FT4 levels and anti-TPO levels. Previous studies also did not demonstrate an association between EAT and anti-TPO levels similarly to our study [28, 31].

The limitations of our study are the cross-sectional nature of the work, small patient numbers, and an absence of information on disease duration.

Consequently, an increase of VAAT in patients with SH is associated with atherosclerosis. WC is an easy metohd to evaluate VAAT but it has low specificity, because of that, to determine VAAT for increased risk of atherosclerosis in these patients. EAT can help because of high correlation with VAAT and simple, low cost, non-invasive method.
